# Supplementary material for: The Role of Partial Enteral Nutrition for Induction of Remission in Crohn’s Disease: A Systematic Review of Controlled Trials
Source: Nutrients. 2022 Dec 9;14(24):5263. doi: 10.3390/nu14245263 (PMC9784970; doi:10.3390/nu14245263)
Supplement: Supplementary file 1 [file nutrients-14-05263-s001.zip › nutrients-2047221-supplementary.pdf]

Supplementary Materials

Table S1. Full-text articles assessed for eligibility but not included in the review.

Reference (year, country)	Type Study	Aims/Intervention	Age	Reason of exclusion
**Y. Kang et al. 2015 [36]**	Clinical trial	To analyze the effect of short-term supportive temporary partial enteral nutrition therapy for treating severe pediatric Crohn's disease (CD)	Children	Type of intervention Concomitant steroids
**P. Sökülmez et al. 2014 [37]**	Randomised clinical trial	To investigate the prevalence of malnutrition in patients with inflammatory bowel disease (IBD) by subjective global assessment (SGA) and the effects of oral nutritional support on the clinical parameters, consumption of energy, macronutrients and fiber intake	Adults	Type of intervention Concomitant steroids
**R. Lev-Tzion R et al. 2021 [38]**	Randomised clinical trial	To evaluate bone formation and resorption in children comparing 6 weeks EEN followed by 6 weeks 25% partial enteral nutrition (PEN) with 6 weeks of 50% PEN with a CD exclusion diet followed by 6 weeks of 25% PEN with exclusion diet	Children	Outcome
**C. Wall et al 2020 [39]**	Clinical trial	To determine whether adherence to EEN was associated with conscientiousness compared with PEN.	Adults	Outcome
**J. Marques et al. 2020 [40]**	Clinical Trial	To investigate the effect of partial enteral nutrition (PEN) on bone health and growth throughout by analysing plasma samples	Children	Type of intervention Maintenance therapy
**A.Brückner et al. 2020 [41]**	Clinical Trial	To investigate the efficacy of partial enteral nutrition (PEN) on bone health, growth and course in CD patients and assessed microbial and metabolic changes induced by PEN	<19 years	Type of intervention Maintenance therapy

**T. Yamamoto et al. 2012 [42]**	Prospective, non-randomized, parallel, controlled study	To investigate the long-term effect of enteral nutrition (EN) as a maintenance therapy in Crohn's disease (CD) patients following surgery	Adults	Type of intervention Maintenance therapy

Table S2. Risk of bias of the selected studies. RoB 2: revised tool for assessing risk of bias in randomized trials.

	**A. Levin et al.**	**D. Urlep et al.**	**C. Wall et al.**	**R. Sigall-Boneh et al.**	**H. Yanai et al.**
**Bias arising from the randomisation process**					
1.1 Was the allocation sequence random?	Yes (Lower risk of bias)	No (Higher risk of bias)	No (Higher risk of bias)	Yes (Lower risk of bias)	Yes (Lower risk of bias)
1.2 Was the allocation sequence concealed until participants were enrolled and assigned to interventions	Yes (Lower risk of bias)	No(Higher risk of bias)	Yes (Lower risk of bias)	Yes (Lower risk of bias)	Yes (Lower risk of bias)
1.3 Did baseline differences between intervention groups suggest a problema with the randomisation process?	No (Lower risk of bias)	No (Lower risk of bias)	No (Lower risk of bias)	No (Lower risk of bias)	No (Lower risk of bias)
Risk-of-bias judgment	*Low*	*High*	*Some concerns*	*Low*	*Low*
**Bias due to deviations from intended interventions**					
2.1 2.1 Were participants aware of their assigned intervention during the trial?	Yes (Higher risk of bias)	Yes (Higher risk of bias)	Yes (Higher risk of bias	Yes (Higher risk of bias)	Yes (Higher risk of bias)
2.2 Were carers and people delivering the interventions aware of participants' assigned intervention during the trial?	Yes (Higher risk of bias)	Yes (Higher risk of bias)	Yes (Higher risk of bias)	Yes (Higher risk of bias)	Yes (Higher risk of bias)
2.3 If Y/PY/NI to 2.1 or 2.2: Were there deviations from the intended intervention that arose because of the trial context?	No (Lower risk of bias)	PY (Higher risk of bias)	Yes (Higher risk of bias)	No (Lower risk of bias)	PN (Lower risk of bias)
2.4 If Y/PY/NI to 2.3: Were these deviations likely to have affected the outcome?	NA	PY (Higher risk of bias)	PY (Higher risk of bias)	NA	NA
2.5 If Y/PY to 2.4: Were these deviations from intended intervention balanced between groups?	NA	Yes (Lower risk of bias)	Yes (Lower risk of bias)	NA	NA
2.6 Was an appropriate analysis used to estimate the effect of assignment to intervention?	Yes (Lower risk of bias)	No (Higher risk of bias)	Yes (Lower risk of bias)	Yes (Lower risk of bias)	Yes (Lower risk of bias)
2.7 If N/PN/NI to 2.6: Was there potential for a substantial impact (on the result) of the failure to analyse participants in the group to which they were randomised?	NA	PN (Lower risk of bias)	PN (Lower risk of bias)	NA	NA
Risk-of-bias judgment	*Some concerns*	*High*	*High*	*Some concerns*	*Some concerns*
**Bias due to missing outcome data**					
3.1 Were data for this outcome available for all, or nearly all, participants randomised??	Yes (Lower risk of bias)	Yes (Lower risk of bias)	Yes (Lower risk of bias)	Yes (Lower risk of bias)	Yes (Lower risk of bias)

3.2 If N/PN/NI to 3.1: Is there evidence that the result was not biased by missing outcome data	NA	NA	NA	NA	NA
3.3 If N/PN to 3.2: Could missingness in the outcome depend on its true value?	NA	NA	NA	NA	NA
3.4 If Y/PY/NI to 3.3: Is it likely that missingness in the outcome depended on its true value?	NA	NA	NA	NA	NA
Risk-of-bias judgment	*Low*	*Low*	*Low*	*Low*	*Low*
**Bias in measurement of the outcome**					
4.1Was the method of measuring the outcome inappropriate??	No (Lower risk of bias)	No (Lower risk of bias)	PN (Lower risk of bias)	No (Lower risk of bias)	No (Lower risk of bias)
4.2 Could measurement or ascertainment of the outcome have differed between intervention groups?	PN (Lower risk of bias)	PN (Lower risk of bias)	PN (Lower risk of bias)	PN (Lower risk of bias)	PN (Lower risk of bias)
4.3 If N/PN/NI to 4.1 and 4.2: Were outcome assessors aware of the intervention received by study participants?	Yes (Higher risk of bias)	Yes (Higher risk of bias)	Yes (Higher risk of bias)	Yes (Higher risk of bias)	Yes (Higher risk of bias)
4.4 If Y/PY/NI to 4.3: Could assessment of the outcome have been influenced by knowledge of intervention received?	PY (Higher risk of bias)	PY (Higher risk of bias)	Yes (Higher risk of bias)	Yes (Higher risk of bias)	PY (Higher risk of bias)
4.5If Y/PY/NI to 4.4: Is it likely that assessment of the outcome was influenced by knowledge of intervention received?	PN (Lower risk of bias)	PN (Lower risk of bias)	PN (Lower risk of bias)	PN (Lower risk of bias)	PN (Lower risk of bias)
Risk-of-bias judgment	*Some concerns*	*Some concerns*	*Some concerns*	*Some concerns*	*Some concerns*
**Bias in selection of the reported result**					
5.1 Were the data that produced this result analysed in accordance with a prespecified analysis plan that was finalised before unblinded outcome data were available for analysis?	Yes (Lower risk of bias)	PY (Lower risk of bias)	PY (Lower risk of bias)	Yes (Lower risk of bias)	Yes (Lower risk of bias)
Is the numerical result being assessed likely to have been selected, on the basis of the results, from:					
5.2 ... multiple eligible outcome measurements (eg, scales, definitions, time points) within the outcome domain?	PN (Lower risk of bias)	PN (Lower risk of bias)	PN (Lower risk of bias)	PN (Lower risk of bias)	PN (Lower risk of bias)
5.3 multiple eligible analyses of the data?	PN (Lower risk of bias)	PN (Lower risk of bias)	PN (Lower risk of bias)	(Lower risk of bias)	PN (Lower risk of bias)
Risk-of-bias judgment	*Low*	*Low*	*Low*	*Low*	*Low*
**Overall bias**					
**Risk-of-bias judgment**	**LOW**	**HIGH**	**HIGH**	**LOW**	**LOW**

PY=probably yes; PN=probably no; NA=not applicable; NI=no information. Adapted from:"RoB 2: revised tool for assessing risk of bias in randomized trials. Table 1" [30].
